# Microwave Sensors for *In Situ* Monitoring of Trace Metals in Polluted Water

**DOI:** 10.3390/s21093147

**Published:** 2021-05-01

**Authors:** Ilaria Frau, Stephen Wylie, Patrick Byrne, Patrizia Onnis, Jeff Cullen, Alex Mason, Olga Korostynska

**Affiliations:** 1 Faculty of Engineering and Technology, Built Environment and Sustainable Technologies (BEST) Research Institute, Liverpool John Moores University, Liverpool L3 3AF, UK; ilariafrau88@live.com (I.F.); S.R.Wylie@ljmu.ac.uk (S.W.); J.D.Cullen@ljmu.ac.uk (J.C.); 2 School of Biological and Environmental Science, Liverpool John Moores University, Liverpool L3 3AF, UK; P.A.Byrne@ljmu.ac.uk (P.B.); p.onnis@exeter.ac.uk (P.O.); 3 Environment & Sustainability Institute and Camborne School of Mines, University of Exeter, Penryn TR10 9FE, UK; 4 Animalia AS, Norwegian Meat and Poultry Research Centre, P.O. Box 396 Økern, 0513 Oslo, Norway; 5 Faculty of Science and Technology, Norwegian University of Life Sciences, 1432 Ås, Norway; alex.mason@nmbu.no; 6 Department of Mechanical, Electronic and Chemical Engineering, Faculty of Technology, Art and Design, Oslo Metropolitan University, 0166 Oslo, Norway

**Keywords:** planar sensors, toxic metals, real-time monitoring, mining-impacted water, water quality, microwave spectroscopy, in situ measurements

## Abstract

Thousands of pollutants are threatening our water supply, putting at risk human and environmental health. Between them, trace metals are of significant concern, due to their high toxicity at low concentrations. Abandoned mining areas are globally one of the major sources of toxic metals. Nowadays, no method can guarantee an immediate response for quantifying these pollutants. In this work, a novel technique based on microwave spectroscopy and planar sensors for in situ real-time monitoring of water quality is described. The sensors were developed to directly probe water samples, and in situ trial measurements were performed in freshwater in four polluted mining areas in the UK. Planar microwave sensors were able to detect the water pollution level with an immediate response specifically depicted at three resonant peaks in the GHz range. To the authors’ best knowledge, this is the first time that planar microwave sensors were tested in situ, demonstrating the ability to use this method for classifying more and less polluted water using a multiple-peak approach.

## 1. Introduction

### 1.1. Water Quality and Trace Metals

Freshwater is an indispensable resource, but it is limited in quantity and quality. Water management is becoming increasingly challenging owing to factors such as climate change, over-exploitation and contamination from both point and diffuse sources due to agricultural and industrial activities [1,2]. Legislation to protect the environment first appeared in the early 1970s, when the European Community and the United States made water quality a priority with the First Environmental Action Programme [3,4] and the Federal Water Pollution Control Act, respectively. Since then, several EU and US directives have been introduced to prevent, monitor, reduce, control and remediate pollution of river basins in Europe and worldwide. Currently, the most important pieces of related water legislation are the European Union Water Framework Directive (EU WFD, 2000/60/EU) and the United States Environmental Protection Agency Clean Water Act (US EPA CWA). They aim to assure good water quality by controlling and limiting contaminants to established standards that are regularly revised [5]. 

Inorganic metals pose a substantial risk to almost half of the water bodies recently monitored in Europe and worldwide. One of the major causes of their dispersion in freshwater bodies is the exploitation of sulphide minerals for the extraction of valuable metals. These include potentially toxic metals, such as zinc (Zn), copper (Cu), lead (Pb) and cadmium (Cd), which are not degraded by normal biogeochemical cycles and can move from one environmental sector to another [6]. They are also accumulated in living organisms including human organs through the food chain [7].

In this work, Cu, Zn, Pb, Cd, etc., are referred to as trace metals due to their toxicity and presence in the environment at generally low concentrations (μg/L range to few mg/L). 

### 1.2. Problem Overview: Mining Areas and Trace Metals Dispersion

Mining activities have been an important contributor to global wealth, but mineral extraction disfigures the landscape and generates huge quantities of waste materials rich in potentially trace metals. They can severely impact the ecosystem and be detrimental to human health [8]. 

The metalliferous veins from which the metals of interest are extracted in non-coal metal mines are mostly sulphide minerals, such as galena (PbS, lead sulphide), sphalerite (ZnS, zinc sulphide), pyrite (FeS_2_, iron sulphide) and chalcopyrite (CuFeS_2_, copper iron sulphide). They are quarried in open pits (removing the surface layer), underground mines (through horizontal tunnels, shafts) or both. The major mechanism associated with the mobilisation of metal ions in mining areas is the oxidation and consequent hydrolysis of sulphide minerals, exposed by mining activity that increases the surface area exposed to weathering and the consequent release of metals [9]. 

This process, which is typically called acid mine drainage (AMD), leads to the dispersion of metal ions in water bodies with high sulphate concentrations and low pH levels (acidic water), well described by the oxidation and dissolution of pyrite [10]. Discharges are not only highly acidic, but they can also be circumneutral. This mainly depends on the two following factors. The first is the ore mineralisation itself, as the oxidation of other sulphide minerals (e.g., galena, sphalerite) does not produce acidity; the second is the neutralisation of the acidity caused by calcium carbonate (if present in the embedding rock). 

Therefore, adequate monitoring and accurate assessment are required to minimise the environmental risk posed by both acidic and neutral mine drainage from active and abandoned mining sites all over the world. 

### 1.3. Abandoned Mines and the Freshwater Environment

**Figure 1:**
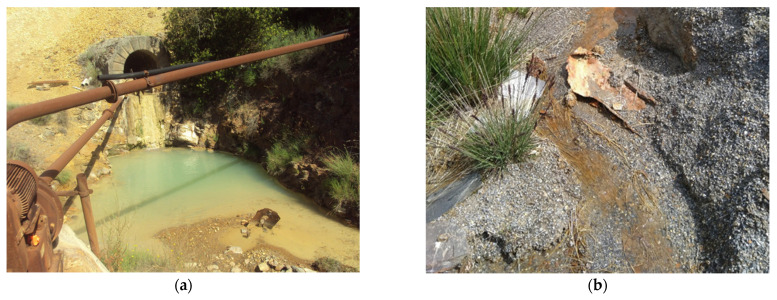
(**a**) Example of a drainage adit in the Montevecchio mine district (south-west Sardinia, Italy); (**b**) leachate from tailings heaps in Nant y Mwyn lead mine (central Wales, UK).

Surface water is the preferential dispersion route of these trace elements even at considerable distances from their source, and several reactions occur in the watershed with the consequent possibility of polluting drinking water supplies. Pollution sources are mainly grouped into two categories: point and diffuse sources. Point sources are mostly drainage adits (Figure 1a), which are groundwater that rises after the pumping used for the mining activity has stopped [11]. Diffuse sources are mainly due to leaching from deposits of waste materials (Figure 1b) either piled up in heaps, along river beds or buried [12]. These trace metal sources are not always easily identified as metals’ mobilisation depends on environmental conditions, such as stream flux, changes in pH and riverbed materials [13,14]. Mobilised metals can also be transported from headwater catchments to coastal areas [15]. 

Consequently, global and European legislation has instituted environmental quality standards (EQS), as “safe concentrations” of trace metals in freshwater, established by the UK Technical Advisory Group on the EU WFD (UK TAG) [16] and US EPA (Environmental Protection Agency) [17] for Zn, Cu, Pb, Cd and sulphates, as well as the metal concentration range for polluted rivers in mining areas worldwide. 

Recently, the Department for Environment, Food and Rural Affairs (DEFRA) has reconsidered standard values by evaluating the pollution above the baseline metal concentration, especially for Zn, recognising the importance of local baseline variability [18,19].

Europe was one of the most productive mining regions in the world. Each country still suffers from water metal pollution problems caused by past mining activity which include rising mine waters (which sometimes intercept important aquifers), and surface water pollution arising from the discharges of spoil heaps [20,21]. In England and Wales alone, there are 4923 abandoned metal mines [22] that pollute water bodies. A total of 9% of rivers in England and Wales, and 2% in Scotland, carry some of the biggest discharges of metals such as zinc, copper, lead and cadmium to the seas around Britain, failing targets for good chemical and ecological status established by the WFD. For instance, Table 1 summarises some examples of polluted water in mining areas with high Zn, Cu and Pb concentrations for some selected countries in Europe (Spain, Italy, Finland, Norway, North Macedonia, Germany) as well as in the United Kingdom, mostly Wales. Generally, in England and Wales, the pH of water is mostly circumneutral (6.5–7.5), as pyrite-based mineralisations are sporadic [22]. An exception is Parys Mountain mine, where the ore is based on chalcopyrite, galena and sphalerite, with abundant pyrite forming a unique deposit in the UK, which produces very acidic conditions, pH of 2–3 in the river basin [23].

It is difficult to identify, characterise and quantify point and diffuse sources of trace metals in polluted mining areas. Metal ions are not “static” but are involved in reactions between water, solid phases and organisms under different geochemical and hydrological settings, in addition to human actions [7]. Currently, water resources in a watershed require sampling at different locations and consequent laboratory analysis of these samples.

### 1.4. Gold Standard Methods for Trace Metals Analysis

The accredited laboratory-based techniques for detecting toxic metals in water include inductively coupled plasma-optical/atomic emission spectrometry (ICP-OES/ICP-AES), inductively coupled plasma-mass spectrometry (ICP-MS) and atomic absorption spectroscopy (AAS) [33]. These methods are highly sensitive and selective, although they need sample preparation, trained staff, expensive disposable equipment and gas for running experiments [34]. These gold standard methods provide off-line monitoring, low-frequency data sampling and delays between sampling and availability of the results. This limits the ability to characterise point and diffuse sources related to metal dynamics when environmental conditions change and to detect an unexpected change in toxic metal pollution as soon as it happens. 

Consequently, worldwide researchers are working on developing novel techniques able to identify and distinguish trace metal ions both qualitatively and quantitatively in situ. Specifically, it is interesting to notice that there is an enormous difference between on-site and in situ measurements. Explicitly, on-site means that the system is portable, although the probe cannot be immersed directly in the water, and samples need to be collected and prepared; instead, in situ means the sensor is probing in the water, and the sample is not collected [35].

### 1.5. State-of-the-Art and Novel Strategies for Trace Metals Analysis

Considering their importance, attention to in situ monitoring systems is increasing, and researchers and industries around the globe are working on finding affordable and effective sensing technologies that can guarantee a rapid response through continuous measurements [36]. During the last two decades, technologies for analysing water quality have evolved, intending to offer the advantages of operational surveillance and early warning in situ. Modern approaches are based on different methods for on-site monitoring including electrochemical, potentiometric, lab-on-chips, optical and biosensors, among others. 

Electrochemical methods are considered the only current sensing systems with high sensitivity and that can be adapted and adopted for on-site monitoring [37]. They are low cost and give a rapid response. They comprise three parts: (1) an electrochemical sensing system; (2) an electrochemical detecting instrument; (3) an electrolyte. The detection device is usually composed of three electrodes: a working electrode (WE), reference electrode (RE) and counter electrode (CE). The modification of the surface of the WE allows the specific identification of selected metal ions [38]. Metal cations are reduced on the working electrode surface and transfer electrons, which generate a measurable signal. The principle is based on the quantification of the metal ions under test depending on a variation in electrical parameters, such as resistance, potential, current or the current–voltage curve. These methods present several advantages, such as high sensitivity, accuracy and speciation determination, although they tend to have low selectivity [39]. 

Ion-selective electrodes (ISEs) convert the activity of ions dissolved in a solution to electrical potential. Depending on the material on their membrane, these are mainly categorised into three groups: polymeric, polycrystalline and glass membrane ISEs. Only the target ions pass through the membrane. They are selective, low cost and portable for in situ monitoring. They are also promising in terms of miniaturisation and integration into standalone sensing units [40], but they are only able to detect a single pre-selected metal ion at a time, and they have limited durability. Additionally, they suffer from interferences from other ions and potential drift after some time. Parat and Pinheiro [41] developed the ISIDORE probe based on the Donnan membrane technique, which was able to determine free Zn, Cd and Pb concentrations in freshwater. This was also promising for in situ monitoring, but they did not proceed with this aim. 

Novel research is evaluating the integration of microfluidic processors and voltammetry, intending to miniaturise the device for toxic metal detection. This is based on microchips, hence “lab-on-a-chip”. These devices are manufactured at a low cost (as they can be paper-based [42]) and, with a diameter of a few centimetres, they are portable. Lab-on-a-chip can enable chemical reactions and can be made to communicate with a smartphone app [43]. Wooseok et al. [44] described a polymer lab-on-a-chip sensor for on-site Pb (II) detection using SWASV. It claims reusability and an environmentally friendly electrode, as it replaces mercury and bismuth, and has high repeatability and a low detection limit (DL). 

Optical sensors are capable of identifying the presence of toxic metals at specific wavelengths in water using conventional methods, such as absorption, reflection or luminescence spectrometry. These sensors can be disposable, such as test strips, or by using optical fibres, capillary-type devices and fluorescent compounds [45], but they suffer from poor selectivity, a high DL and reversibility. As with electrochemical sensors, optical devices can also be integrated with lab-on-a-chip (microfluidic) devices. Fibre optic sensors can give fast and accurate responses. Optical fibres consist of cores and claddings with a different reflection index. They are connected to a light source and a light beam travels through it and produces an optical response of the target. The monitoring of toxic metals simultaneously in water was not investigated fully until recent years. Lately, Kopitzke and Geissinger [46] developed a novel optical fibre sensor array with the inclusion of a fluorescent compound for Cu and Zn detection which gives fast and accurate results (RSD 10%), with high sensitivity and selectivity (DL of sub-ppm). Further, Halkare et al. [47] experimented with the integration of bacteria (*E. coli B40*) on nanoparticles and obtained a fast response (10 min) with a much lower DL (0.5 ppb) for transitional metals, although the selectivity was only proven by comparing Cd and Hg, which are chemically quite different.

In recent years, biosensors have been widely investigated for detecting toxic metals in water. They are constituted by the integration of sensitive biological components, such as enzymes, nucleic acids, bacteria, antibodies, antigens, etc., on a sensing structure [48]. These biological elements interact or bind with a specific analyte under test. Their main advantage is the ability to measure bioavailability [49]. The transducer can be optical, electrochemical or electroluminescent, for example. Eltzov et al. [50] produced a new portable whole-cell biosensor for detecting water toxicity. The prototype is integrated with two systems: non-disposable (optoelectronic instrumentation) and disposable (bioluminescent bacteria immobilised in calcium alginate matrix pads) parts. Different toxic pollutants, including Cu and Zn, were detected with the prototype in the laboratory and on-site. The findings showed a highly sensitive response to some of the tested contaminants. This device is attractive due to its ease of maintenance, measuring procedures, portability and sensitivity. Although its sensitivity (ppm range) is too high for detecting Cu and Zn in mining-impacted waters, it is disposable and feasible for on-site monitoring, but not for in situ.

Another technique described by Iqbal et al. [51] is promising for online monitoring of toxic metals in water. This method is based on near-infrared diffuse reflectance spectroscopy (NIDRS) and chemometric detection. This is a rapid and cost-effective technique, although it requires a large sample volume (1 L) and has poor selectivity, which can be, in part, overcome by applying partial least square (PLS) regression models. 

**Figure 2:**
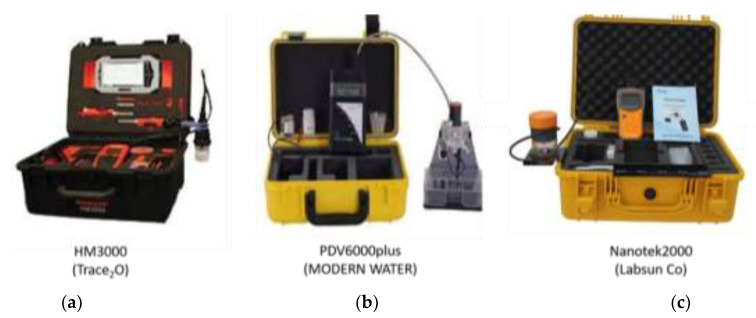
Available toxic metal analysers for on-site monitoring: (**a**) HM3000 form Trace_2_O, Berkshire, United Kingdom; (**b**) PDV6000 plus from MODERN WATER, London, United Kingdom; (**c**) Nanotek2000 from Lubsun Co, Shaanxi, China.

Commercially available electrochemical devices are capable of on-site monitoring (as they can be portable), but they require sample collection, which makes them unsuitable for continuous in situ measurements (probing the water) and for detecting variations in water contamination. Among the few commercial products, the most efficient portable systems are the Metalyser^®^ Portable HM1000 and HM3000 (from Trace_2_O, Figure 2a), the PDV 6000 plus (from MODERN WATER, Figure 2b), the Nanotek2000 (from Labsun Co, Figure 2c) and HM-3000P (from Skyray Instruments), which are based on voltammetry principles [52]. Thus, they have a low DL (ppb), can be used on-site but not in situ, need sample preparations and are not able to detect multi-metals simultaneously (only two metals simultaneously) in 5–10 min [53]. 

In addition, these instruments are large and arguably too expensive to be deployed as part of a monitoring network. Some other available portable cheap options are given by analysers based on the colourimetric principle [54]. 

No single system available today can fully meet the need to determine, in real time, the composition of water to the desired sensitivity level and cost for long-term monitoring of water bodies affected by metal mine drainage. 

In this work, the initial development and first in situ testing of a novel sensing system based on microwave spectroscopy for detecting, in real time, the contamination level of polluted freshwater by mining activities are described.

## 2. Microwave Spectroscopy

### 2.1. Sensing at Microwave Frequencies

Spectroscopy methods are widely used in analytical chemistry. The absorption or transmission of the EM radiation at specific frequencies or wavelengths can be related to the structure or concentration of a gas, liquid or solid material. For sensing, specific spectroscopy methods are used depending on (i) the frequency or wavelength; (ii) the form of the material under test; (iii) the sensing purpose, such as ionic, elemental composition or molecular determination. The nature of the interaction depends on the energy of the radiation. Analysing the EM spectrum from high to low energies, gamma and X-ray radiations break chemical bonds; ultraviolet radiations cause transitions between electronic energy states within a molecule; infrared and Raman cause internal vibrations within the molecule; and microwaves cause molecules to rotate. The microwave output is similar to other spectra that are measured, but it operates at the GHz frequency range and low energy.

Using EM waves at microwave frequencies for sensing purposes is an active research approach with the potential for commercialisation. This novel sensing approach has several advantages, including non-invasiveness, non-destructiveness, immediate response when the EM waves are in contact with a material under test (MUT), low cost and power, providing the opportunity to guarantee continuous monitoring of freshwater resources. 

Ongoing research on microwave spectroscopy has recently demonstrated the ability to detect changes in many materials, thanks to the adaptability of the sensing structure. During the last three decades, microwave spectroscopy for liquid sensing has been investigated. However, measurements of liquids are complex and not fully understood, as the rotation is hindered by intermolecular forces, and the bandwidths are much greater. The microwave is an oscillating electromagnetic field and if the molecule is polar, the microwave field can couple with the molecular dipole and cause it to rotate, but rotation is hindered so the molecule cannot re-orientate fast enough to follow the field reversals exactly. Thus, it loses energy which appears as heat. Fundamentally, there are two components to consider: (i) the dielectric permittivity and (ii) the dielectric loss [55]. 

The principle of microwave spectroscopy is based on the singular interaction between incident waves at specific frequencies and the analyte presented to the sensing structure. The change in the spectral response at specific frequencies depends on variations in permittivity and/or conductivity, which can be linked to the composition and concentration of the measured solution [56]. Conductivity alone is not sufficient to explain the variations in complex permittivity [57]. Accordingly, permittivity (*ε_r_*), as defined in Equation (1), relates to a material’s ability to transmit an electric field and is a complex value which varies with changing frequency and temperature, accounting for both the energy stored by a material (*ε*′), which indicates the ability to be polarised by the external electric field, and any losses of energy that occur (*ε*″), which quantify the efficiency with which the electromagnetic energy is converted to heat.

*ε_r_* = *ε′* − *jε″* (1)

The response of the sensor manifests itself as a resonant frequency change or an attenuation of the signal [58]. Different materials have diverse permittivities, and a mixture has a permittivity value which depends on the permittivity of each component and its structure [59]. The correlation of the permittivity of a material with its composition can indicate the properties of the material, with a consequent identification of the changes in the material’s parameter. 

**Figure 3:**
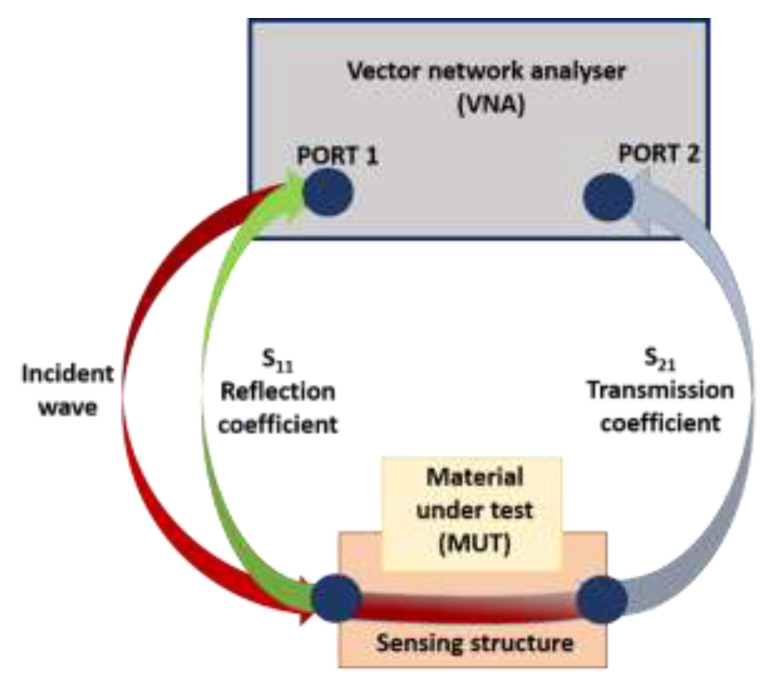
Sketch of measurement set-up and output (S_11_ and S_21_).

The principle of using microwave spectroscopy is based on the interaction of electromagnetic waves with the tested sample through a sensing structure. The measurement is based on the unique interaction between EM waves at microwave frequencies and a sample. The source is a vector network analyser (VNA), which provides a stimulus at low power (<1 mW) and monitors the response as S-parameters (S_nn_, scattering parameters), which use matched loads (50 Ω) to characterise EM behaviour. A VNA can be configured with one or two ports (Figure 3). 

A one-port configuration (S_11_ measurement) measures the reflection coefficient (return loss or Γ) of an MUT, which depends on how much the incident wave propagates through or is reflected by the sample. A two-port configuration (S_21_ measurement) allows the measurement of the transmission coefficient, which depends on how much EM power propagates from one port (port 1) through the MUT and is received at the second port (port 2). This configuration allows the determination of both transmitted and reflected signals. S-parameters vary with frequency. The output has an amplitude and a phase, so it is a vector quantity. Amplitude (reflection coefficient magnitude, |S_11_|) is shown as a spectral response in dB (*y*-axis) versus frequencies (*x*-axis) and represents the amount of energy that is absorbed at that specific frequency. Changes in the spectral output can also be related to changes in impedance parameters, such as resistance (R) and capacitance (C). As the frequency increases, voltage and current become harder to define because the wavelength becomes small compared to the circuit dimensions. The reflection coefficient magnitude (|S_11_|, described as S_11_ in this work) then becomes a more useful representation. A reflection coefficient of 0 dB represents a mismatch (100% reflection); an S_11_ of-∞ dB is a perfect match (0% reflection/100% transmission).

By investigating the EM spectral response, it is possible to identify specific variations related to the MUT. However, the response is also dependent on variations in other parameters, such as temperature, density and pressure, that can be overcome by understanding the causes and interferences in the output signal. 

In a simple case of two components in an MUT, it is possible to presume the variations in S_11_ which are related to one of the two components [59]. However, if the sample under test is complex and variations are related to more constituents in the sample, more research and deeper signal analysis are necessary for clearer identification. The simultaneous existence of multiple variables such as temperature, density, moisture and structure will affect the microwave response.

### 2.2. Microwave Sensors: Applications and Versatility 

Microwave spectroscopy can give an immediate response as soon as a sample is in contact with the EM through a sensing structure. Consequently, microwave spectroscopy has emerged in recent years as a novel monitoring technique in the food industry [60,61], healthcare [62], sports science [63], built environment [64], structural analysis [65], environmental monitoring [66] and water quality control [67]. One reason that makes this method highly adaptable is the various physical forms that the sensing structure can take: resonant cavities, waveguides, horn antennas, flexible and planar resonant sensors, depending on the form of MUT. For the analysis of liquids, waveguides and horn antennas are not generally suitable, so resonant cavities and planar sensors are more common. 

### 2.3. Sensor Types: Resonant Cavities and Planar Sensors for Liquid Detection

Current research [68] has demonstrated the possibility of identifying the presence, and quantifying the concentration, of specific components in water including a mixture of water and other liquids (e.g., water and alcohol, water and fuel). In recent decades, research has been carried out to measure liquid materials using microwave spectroscopy. Considering the variability of the sensing structures, the most successful experiments for detecting a mixture of diverse liquids (e.g., oil and water) or target particles in liquids were obtained using resonant cavities and planar sensors. 

Several experiments have shown resonant cavities to be able to detect the presence and concentration of various materials in liquid under test (LUT). Table 2 summarises some examples of work that has been performed for measuring the composition and concentration of liquid materials. 

Despite the success of using resonant cavities for liquid measurements, they are not practical for in situ monitoring of polluted freshwater, as they cannot directly probe water. A sample needs to be collected and placed inside the resonant cavity for being analysed. A possible solution to this problem is the integration of fluidic channels where the water sample is pumped through the sensor, as in the substrate integrated waveguide developed by Wei et al. [74] for acetone and water mixtures. Recently, Andria et al. [75] designed and modelled a coaxial structure for the real-time measurement of water-in-fuel for the automotive field. 

On the other hand, planar sensors are a cost-effective and practical option for in situ and long-term continuous measurements of freshwater, being able to directly probe the water. Between the numerous possible resonant structures, planar sensors have the potential to give high sensitivity and accuracy [76]. They have the advantages of small size, robustness and low-price fabrication. They are light and practical for in situ and continuous monitoring. They can be rigid [67] or flexible [77] and soldered with SMA connectors, for connecting to a coaxial cable. 

In recent years, several planar microwave sensors with different conformations have been developed and tested for diverse liquid sample compositions in deionised water (DW) and various mixtures, for both qualitative and quantitative concentration measurements. Some examples are summarised in Table 3.

Summarising, most of the tested microwave resonant structures (both resonant cavities and planar sensors) were tested for nitrates, chlorides and various alcohol mixtures, among others. Therefore, the feasibility to quantify various particles in water and matrix components (e.g., water, oil, alcohol) at specific frequencies of the EM spectrum has been demonstrated. 

### 2.4. Microwave Sensors and Trace Metals Analysis

A proof of concept that demonstrated the feasibility of detecting Pb^+2^ ions in DW using microwave spectroscopy and a resonant cavity was provided by Korostynska et al. [90]. Considering its impracticability for in situ monitoring, this work initially demonstrated the detection of Pb^+2^ ions using planar sensors at high concentration (1–100 mg/L). The successful action of the resonant structure demonstrates the real-time ability to detect changes in Pb concentration (0, 1, 10, 50 and 100 mg/L), placed in 50 mL centrifuge tubes, with a good linear correlation, with R^2^ = 0.9527 and R^2^ = 0.9017, respectively, at two frequencies, 415 MHz and 2.45 GHz, after processing of the raw data. This experiment demonstrates the feasibility to have an inexpensive real-time detection of Pb at various concentrations as soon as the EM waves interact with the water sample under test. Consequently, planar Au eight-pair IDEs onto PTFE substrates were tested by Frau et al. [91] with metal water solutions at the same concentrations for evaluating the feasibility to detect, in real time, metal concentration variations using a smaller and more suitable resonant structure for consequent in situ measurement. After each measurement, responses returned to the original position (air spectra), confirming that the developed resonant cavity and microwave sensors are reliable and reusable, and thus a sustainable solution for continuous water quality monitoring. As the concentrations used in this experiment were too high, smaller concentrations were consequently investigated for the real metal concentration of Cu and Zn that can be commonly found in mining-impacted water [92,93].

After having evaluated the possibility of measuring changes in metal concentrations using low-cost planar IDE sensors and having assessed the comparable response obtained with the resonant cavity, these sensors were selected for additional experiments.

Puangngernmak and Chalermwisutkul [94] are the only researchers who also experimented the detection of trace metals (Cu, Zn and Ni) in water with concentrations of 1, 10, 100 and 1000 mg/L using an open-ended coaxial structure and a VNA. They demonstrated the detection of these metals at frequencies lower than 2 GHz and the differentiation of these metals between 2 and 3 GHz. Notwithstanding, they showed the differentiation of only high concentrations (100 mg/L) of these metals, resulting in the system not being adequate for the detection of these metals in mining-impacted waters.

The aim of this work is to further investigate the feasibility to detect, in real time, trace metals (Cu and Zn) in mining-impacted water, in both the laboratory and in situ, for common metal concentrations found in mining-impacted areas. Planar sensors were adapted to directly probe freshwater, and the ability to detect, in situ and in real time, the pollution level in four mining areas in the UK was investigated for the first time.

## 3. Materials and Methods

### 3.1. Water Samples

**Figure 4:**
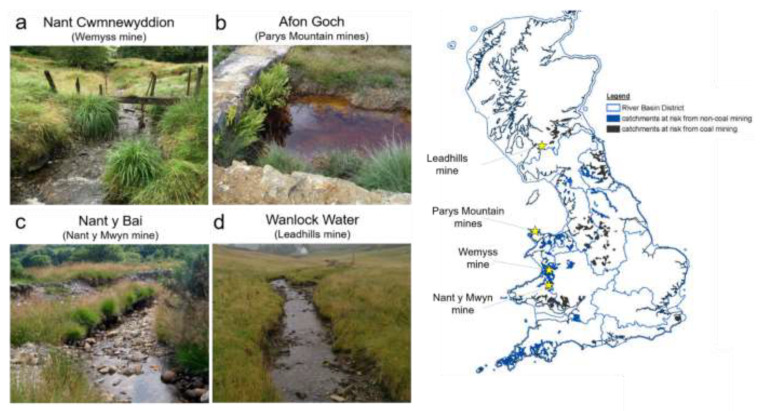
Example of polluted streams in the UK from where samples were collected and tested in the laboratory and in situ using microwave spectroscopy: Nant Cwmnewyddion (**a**), Afon Goch (**b**), Nant y Bai (**c**) (Wales) and Wanlock Water (**d**) (Scotland); the map is adapted from the Environment Agency [11] which shows catchments at risk from non-coal (in blue) and coal (in black) mines and highlights the four mining areas with yellow stars.

Following the successful testing of “simple” laboratory-prepared samples (e.g., mono-metal polluted, such as Zn and Cu) [93,95], in this work, mining-impacted waters were sampled and analysed. Specifically, various freshwater samples were collected from four polluted mining areas in the UK: three in Wales and one in Scotland (Figure 4). These mining areas are as follows:

(a) Wemyss mine (Mid Wales, UK);
(b) Parys Mountain mining district (Anglesey, North Wales, UK);
(c) Nant y Mwyn mine (Mid Wales, UK);
(d) Leadhills (Scotland, UK).

These mining areas were selected as test sites because they represent the typical trace metal pollution range (very high, average and low) found in the UK. Specifically, Parys Mountain mining district represents an extremely polluted site, with Cu and Zn concentrations >20 mg/L; Wemyss and Nant y Mwyn mines represent “averagely” polluted sites in the UK, with a Zn concentration ranging from 0.8 to 9 mg/L; and Leadhills mine is a low polluted site, with metal concentrations just above the EQS, with 0.1–0.3 mg/L of Zn.

Specifically, a sample was collected in the Nant Cwmnewyddion stream in the Wemyss mine area (acronym: NC). Four samples were collected in the Parys Mountain mining district, specifically the Dyffryn Adda Adit (acronym: PM (A)) and two other samples in the mining area (acronyms: PM-1 and PM-2), and one after the wetlands (PM-W). Three samples were collected: two along the Nant y Bai stream (acronyms: NYB-1 and NYB-2) and a right bank inflow, a run-off from tailings deposited on the riverside (acronym: NYB-R). A sample downstream of Wanlock Water (acronym: WW-1) was collected. In these mining-impacted streams, in situ and continuous measurements were performed using planar sensors probing the water. Some samples were spiked using the standard addition method and certified Cu and Zn 1000 ppm ICP standard solutions (from Sigma-Aldrich, respectively, 18,562 and 68,921) for evaluating calibration curves, as described by [95].

For all the samples, physicochemical parameters (pH, EC, T) were measured after appropriate calibration using a multi-parameter meter (model PCE-PHD 1, PCE Instruments). The EC was calibrated using a standard solution of 1413 µS/cm, which was corrected for temperature; the pH was calibrated using pH calibration solutions 4, 7 and 10. The temperature was also constantly monitored using a digital and a non-contact infrared thermometer (model TM-902C Lutron and 830-T2 Testo, respectively). The effective concentrations of the samples were analysed using an ICP-MS, model 7900 Agilent Technologies (for low concentrations of cations), and an ICP-OES, model iCAP 6500 Duo Thermo Scientific (for high concentrations, major cations and/or higher metal concentrations), both equipped with an auto-sampler. Samples for ICP analysis were acidified to 1% *v*/*v* with high-purity (>67%) HNO_3_.

### 3.2. Sensors and Measurement Development

**Figure 5:**
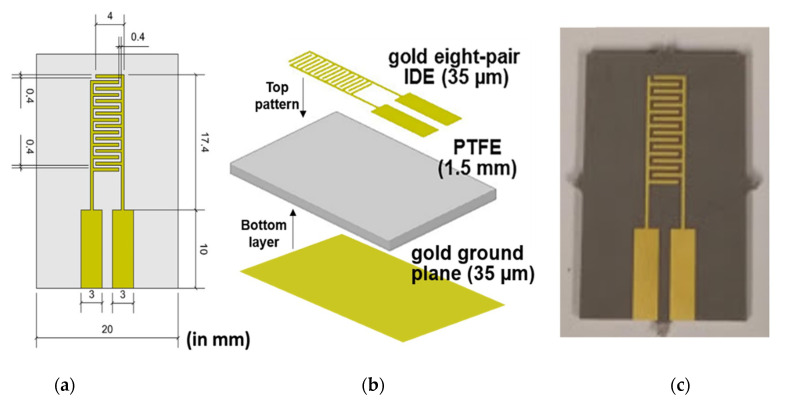
(**a**) Scheme with size of an Au eight-pair IDE sensor (mm) showing its front view, (**b**) 3D view and (**c**) a picture of it.

Gold-plated (Au) eight-pair IDE microwave sensors on PTFE substrates (Figure 5a–c) were selected as planar sensors for measuring the variation in metal concentration in polluted water, in both the laboratory and in situ. The layout and dimensions of the eight-pair IDE pattern sensor are shown in Figure 5a. Gold was used as the conductive metal material for both the bottom layer, which acted as a ground plane, and the top pattern to maintain chemical neutrality when the device is placed in contact with the analyte solution. The thickness of the Au layers was 35 μm. The microwave sensor was designed on a 1.5-mm-thick PTFE substrate. A distinct feature of IDE-type sensors is their higher sensitivity to change close to the sensor surface, which reduces the variation due to the external environment [83].

Some sensors were also covered with a PCB lacquer spray coating for electrical circuit protection and to avoid oxidation of the gold electrodes.

**Figure 6:**
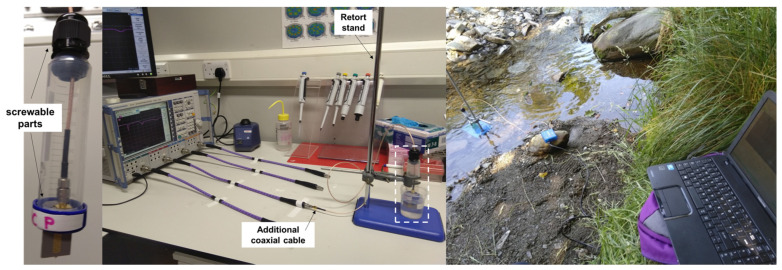
(**a**) Close up of the sensor and its structure with screwable parts; (**b**) measurement configuration adopted using a ZVA 24 configured with an additional coaxial cable and a retort stand for holding the waterproofed microwave sensor in place probing a water sample; (**c**) configuration for *in situ* measurements using a miniVNA tiny and a laptop as output device.

Microwave sensors were adapted for directly probing the water for in situ monitoring. Recently, Reyes-Vera et al. [96] developed a submersible permittivity sensor for liquid monitoring. For this purpose, the IDE sensors were waterproofed using a thermoplastic adhesive, consisting of ethylene-vinyl acetate (EVA) and terpene-phenol resin (TPR) (internal part), and silicone (external part), and embedded in a specific structure adapted from 50 mL centrifuge tubes, which allowed access for fastening the selected sensing structure and for tightening/untightening the coaxial cable in 50 mL centrifuge tube lids, Figure 6a, as it was previously tested by [95]. After initial data analysis and frequency selection, the S_11_ response (reflection coefficient magnitude) was recorded continuously (n = 5) between 10 MHz and 3 GHz using a Rohde and Schwarz ZVA 24 VNA (with 60,000 discrete points) and a miniVNA tiny (Mini Radio Solutions) (with 1000 discrete points), through coaxial cables with the configuration shown in Figure 6b,c, respectively, for laboratory and in situ measurements. The waterproofed sensor was held in position by a retort stand, in a 40 mL water sample and using an additional coaxial cable. The calibration of the ZVA 24 VNA and the miniVNA tiny was performed on both cables to delete its effect.

The low-cost miniVNA tiny was selected to perform *in situ* measurements, due to its simplicity and practicability. The miniVNA tiny is capable of sweeping between 1 MHz and 3 GHz with unit dimensions of approximately 80 × 80 × 35 mm. It has an SMA-style connection on one face for DUT and DET (equivalent to one-port and two-port configurations, respectively) and a USB connection on the rear. It operates via the USB connection, requiring 5 V, is connected to a laptop and uses *VNA*/*J* as data acquisition software. In addition, it was able to continuously save the data response every 5–10 s. Measurements of mining-impacted water were performed to assess (i) the prospect for quantifying metal concentrations *in situ* and (ii) the stability of the sensing response with the river flow.

#### Continuous *In Situ* Measurements

The ability to detect an unexpected change in freshwater and then return to the baseline level could not be investigated by injecting trace metals into natural water, so it was evaluated using *slug injections* of sodium chloride (NaCl) as a tracer, usually used for flow measurement evaluations. The tracer is injected into the stream as a near-instantaneous slug [97], named slug injection [98,99] or salt gulp injection dilution gauging [19,27]. A certain amount of salt (e.g., 100–500 g depending on the flow) is mixed in a specific water volume (e.g., 10 L of the same surveyed stream water) in a bucket and injected in a point along the stream. Then, the tracer concentration is measured at a downstream point (e.g., 30–100 m), where the tracer has become uniformly mixed with the streamflow. Equations based on the mass balance principle are then applied to calculate the stream discharge [97]. 

**Figure 7:**
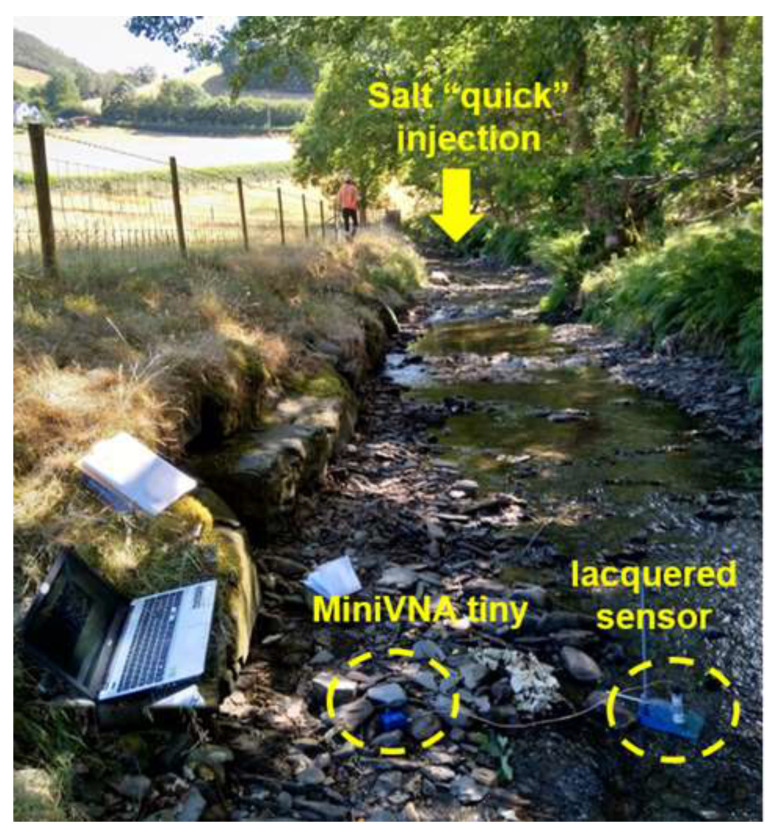
Evaluation of the feasibility to measure “unexpected” variation in a parameter (NaCl in this case) in freshwater and the return to the baseline level.

The feasibility to continuously detect the change in the microwave spectrum with NaCl and then return to its baseline spectrum was investigated (during fieldwork carried out by Onnis, Byrne, Hudson-Edwards, Stott and Hunt [99]) using the miniVNA tiny and a lacquered sensor, connected via USB to a laptop, and extracting data every second (Figure 7).

### 3.3. Data Analysis

Results obtained using the described approach were used as an indicator of metal content. They were analysed using Microsoft Excel and OriginPro9. By studying the microwave responses (S_11_) at specific frequencies, it is possible to evaluate correlations with Cu and Zn. Moreover, the response of multiple peaks was combined for achieving a more specific response. 

Best-fit curves for Zn and Cu concentrations on samples spiked using the standard addition method at specific frequencies of the EM spectrum were evaluated analysing various parameters, including the R^2^ (the square of the Pearson correlation coefficient), the coefficient of variation (CV), which is the ratio of the standard deviation (SD) to the mean, and the sensitivity for every 1 mg/L change in metal content. Specifically, the R^2^ is used for evaluating the correlation (mostly linear) between the spectral response (e.g., S_11_ value in dB) at specific frequencies and the concentration (mg/L) of the metal under test. This allows the development of a calibration curve for evaluating the “unknown” concentration of a sample under test. The CV is used to evaluate the precision of the sensing response and this is achieved by performing repetitive measurements (5–10 times) of the same sample. The sensitivity describes how much the signal changes for a small increase (e.g., for each mg/L) in the metal concentration. It is equivalent to the slope of the calibration curve attained as S_11_ versus the metal concentration [100].

First of all, the mean, the SD and the CV were evaluated. The data with a CV > 5% were not considered, as the response is not repeatable. Then, the spectra for diverse samples were compared. Samples with the same metal (e.g., Cu) at various concentrations (between 0 and 10 mg/L) were used to identify the resonant frequency and the sensitivity. For these, the peaks that produce a higher sensitivity and R^2^ and a lower CV were selected. 

## 4. Results and Discussion

### 4.1. Preliminary Laboratory Analysis Probing Water Samples

**Figure 8:**
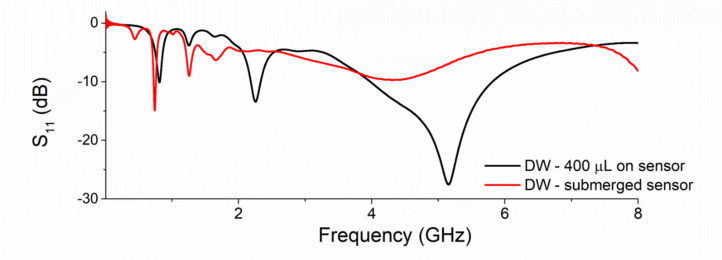
Comparison of the microwave signal between placing 400 µL of water sample onto sensor versus dipping the sensor in a water sample.

Initial experiments demonstrated the feasibility of measuring water samples by dipping the waterproofed sensors in water samples. Figure 8 compares the signal response measured by placing 400 µL of the water sample onto the sensor (black line) (as described by [93]) and submerging the sensors into a DW sample (red line) using the adapted eight-pair IDE sensor directly probing the water sample, keeping in mind the possibility to move to the real-world environment and performing *in situ* measurements.

The signal response changes from using the 400 µL method described in previous works, probably due to the propagation of the EM waves in a diverse volume. As the microwaves propagate in a different volume, the resonant peaks are produced at different frequencies until they produce a stable response once the volume is large enough for the waves to be saturated. With this described configuration, three resonant frequencies were selected between 0.10 and 3 GHz which represent the variation in the metal concentration in the water samples. Similar experiments by submerging planar sensors in the samples were performed by [96] for different liquids (acetone, propyl alcohol, methanol).

### 4.2. Feasibility of Measuring the Increase in Metal Concentration in Collected Water Samples

**Figure 9:**
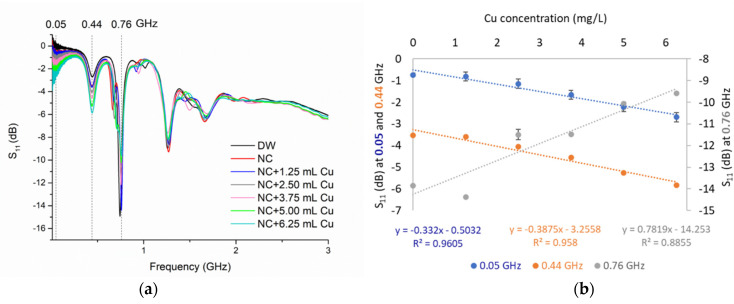
(**a**) Microwave spectral response between 0.01 and 3 GHz for a collected water sample (NC) spiked with standard Cu addition with highlighted (dash lines) resonant frequencies at 0.05, 0.44 and 0.76 GHz; (**b**) linear correlations between the increase in Cu and change in S_11_ response at the three resonant frequencies.

The selected sensors were immersed in the spiked Cu and Zn samples and initially measured using the ZVA 24 in the laboratory. The microwave response was able to determine changes in the metal content, for both metals, with similar reflection coefficients at the same frequencies, due to the similarity of these metals, as previously described by [95]. An example of spectral output between 0 and 3 GHz is illustrated in Figure 9a for a collected sample in Wemyss mine (NC), which was spiked with Cu standard additions (+1.25 mL of Cu). The best linear responses (Figure 9b) were identified at low frequencies (at 0.05 and 0.44 GHz) using this measurement configuration.

Between them, the peak located at 0.44 GHz was selected for its higher linear correlation (R^2^ > 0.96), higher sensitivity (0.38 dB for each 1 mg/L variation in Cu concentration) and low SD (RSD < 0.51%) compared with the other two peaks (Table 4). Although the measurements at 0.76 GHz produce a lower dB (~14 dB) for a low concentration and higher sensitivity, the linearity is poorer as the concentration increases compared with the other two selected peaks. This is due to a higher change in dB for concentrations between 0 and 2.5 mg/L, compared with the higher concentrations (between 3.75 and 6.25 mg/L). Consequently, it is valuable to use a multi-peak approach to benefit from different statistical parameters at specific frequencies.

Notably, the resonant peaks that were produced at frequencies < 0.5 GHz have a lower dB as the metal concentration increases (the reflection decreases) compared to the peaks at frequencies > 0.5 GHz, where, inversely, a higher dB is noticed as the concentration increases (the reflection increases). A multi-peak approach combining peaks with diverse trends is able to provide a more reliable response in determining a specific spectral pattern for specific pollutants. The changes in trend at specific frequencies reflect the changes in the dielectric properties of the material under test with variation in the reflection coefficient signal at specific frequencies. This laboratory experiment demonstrates the feasibility of the microwave response to detect and quantify the increase in metal content in the collected water sample by directly probing the water sample under test. 

By directly submerging the samples, by waterproofing the SMA connectors, in water, valuable achievements were noticed comparing the response of placing the water samples onto the sensor, as described by [93], such as (i) the ability to move into the field and perform *in situ* measurements; (ii) the smaller price of the necessary electronics for developing a portable device using a mini-circuit board, considering the peak locations at lower frequencies; (iii) the improvement in sensitivity at low frequencies compared with the method performed by adding the sample (400 µL) onto the sensor; (iv) the elimination of the peak located at 2.4–2.5 GHz, which can interfere with the wireless communication largely used at that frequency for sending data. Another advantage is the sensitivity improvement: comparing these results, the slope increases with a consequent improvement in the ability to detect lower metal concentrations at lower frequencies with a lower CV. Specifically, for example, there is a sensitivity improvement from 0.065 for each 1 mg/L variation at 2.46 GHz to 0.38 for each 1 mg/L at 0.44 GHz comparing the “Small sample volume onto sensor” method with the “Submerged sensor in water”.

### 4.3. Measurement of Various Water Samples

After having evaluated the feasibility of measuring the increase in metal concentration (Cu) in a collected water sample, different water samples from the previously described mining areas were measured in the laboratory using the ZVA 24. Physicochemical parameters (EC, pH and T) were measured in the field and the laboratory, and results are shown in Table A1, Appendix A. Metal concentrations (Zn, Cu, Pb, Cd, Fe and Mn) were measured using ICP-MS and/or ICP-OES and results are also shown in Table A1, Appendix A.

**Figure 10:**
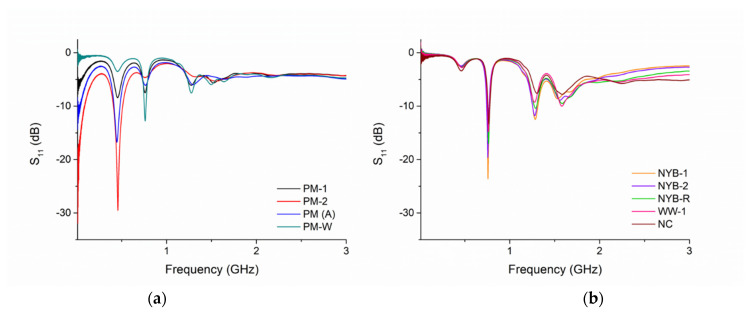
(**a**) Spectral responses captured in the laboratory using a ZVA 24 for samples collected at Parys Mountain (**b**) and in the other three less polluted mining sites, Nant y Mwyn, Leadhills and Wemyss mines.

Figure 10a (for Parys Mountain mining water samples) and Figure 10b (for the three other less polluted mining sites) show the spectral response for collected mining-impacted waters that were analysed in the laboratory using the ZVA 24, at constant temperature (19.0 ± 0.2 °C). 

The sensor was able to classify the pollution level in the water samples tested in the laboratory, after collection. Summarising, the contamination level by area can be generalised, from most to least polluted, as follows:

- Parys Mountain > Wemyss > Nant y Mwyn > Leadhills.

Specifically, the generalised “contamination” level for some of the polluted water samples from two surveyed mining areas (Appendix A) can be represented as follows:

- Parys Mountain mining district: PM-2 > PM-1 > PM (A) > PM-W
- Nant y Mwyn mine: NYB-R > NYB-1 > NYB-2

The microwave sensor was able to measure and distinguish between waters in various mining areas around the UK. In Parys Mountain mine (Figure 10a), PM-2 was the most polluted sample, followed by the adit (PM (A)), and PM-1. The sample PM-W was collected after the wetlands and was less polluted. Accordingly, recent studies have demonstrated a strong reduction in the contamination downstream in the Southern Afon Goch due to natural wetlands [28], which reduce the metal contamination and increase the pH. Natural wetlands naturally promote the reduction in contamination in water, as demonstrated in other mining areas in Europe, such as Rio San Giorgio in the Monteponi mine district (Sardinia, Italy) [101]. 

As it has previously been described, the contamination levels at the peaks located at <0.5 GHz (0.05 and 0.44 GHz) have a lower reflection coefficient S_11_ for higher metal concentrations; controversially, peaks located at > 0.5 GHz (0.76 GHz) have a higher S_11_ for low concentrations. This is well represented for the four samples collected from Parys Mountain mine and analysed in the laboratory (Figure 10a). The less polluted samples from the other three mining areas (Figure 10b) follow the same pattern, despite the fact that more variation is caused by the different water matrix, and by the low concentration of the samples. Notably, the sensors do not resonate at 50 MHz for low metal concentrations. The combination of the S_11_ response at multiple frequencies with diverse trends guarantees a more reliable response with the identification of the pattern recognition for the analysed metal content in water.

Considering the complexity of the analysed mining-impacted water samples, which contained a mixture of various metal concentrations, such as Zn, Cu, and Pb, and the variation in other water parameters, there was not an identical correspondence between the measured concentration using the accredited method (e.g., ICP) and S_11_ response. Consequently, there was no possibility to fit data in the previous calibration curves and quantify the concentration, although the samples follow the same trend.

### 4.4. In Situ Trial Measurements Using a Portable Inexpensive VNA

After having evaluated the feasibility of detecting and distinguishing different water samples from the same and different mining areas in the laboratory, eight-pair IDE sensors were tested in the field using the portable and practical miniVNA tiny, connected to a smartphone or a laptop. *In situ* microwave measurements are compared for evaluating the possibility of differentiating between contaminated samples and, consequently, for prioritising remediation actions. 

**Figure 11:**
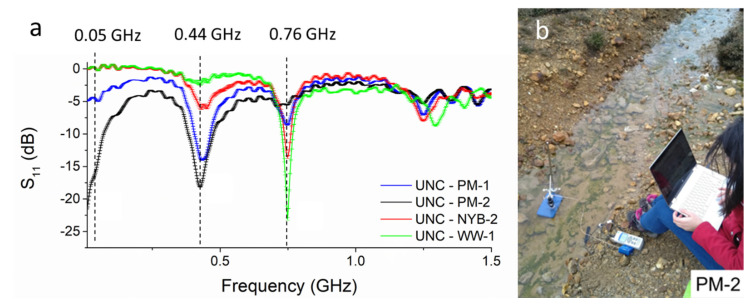
(**a**) Results for *in situ* measurements using a miniVNA tiny and eight-pair IDE sensors probing 4 water samples in three mining areas around the UK; (**b**) shows the most polluted water measured in this study (PM-2) and the measurement configuration.

The sensors were able to qualify and differentiate mining-impacted waters *in situ*. As it has previously been assessed, the microwave sensor at 50 MHz is able to detect high metal concentrations (Figure 11a). The sensor resonates at this frequency only for the samples analysed at Parys Mountain mining district, which suffer from severe contamination, with PM-1 with 12.5 mg/L and 13.5 mg/L and PM-2 (Figure 11b) with 7.0 mg/L and 25.0 mg/L of Zn and Cu, respectively. Further, both have a high concentration of Pb, Cu, Fe and Mn. The responses for peaks at 0.44 and 0.76 GHz reflect the principle previously assessed: a lower dB at 0.44 GHz corresponds to a higher concentration; a lower dB at 0.76 GHz corresponds to a lower concentration. The results shown in these graphs follow the contamination level in the samples: PM-2 > PM-1 > NYB-1 and WW-1. The potential of using microwave spectroscopy and IDE sensors for classifying and prioritising polluted water was assessed, *in situ* and in real time. 

Some variation in the spectral response was assessed comparing the sensing response with the previous laboratory measurements for the same samples, probably caused by (1) the variation in temperature; (2) the imprecise response given by the miniVNA tiny; and (3) other particles in the water. The lower frequency sweep rate of the miniVNA tiny compared with the ZVA 24, 1000 versus 60,000 distinct points between 10 MHz and 3 GHz, produces a spectral response with lower resolution, as it is visible comparing Figure 11a with Figure 10a,b. However, the resonant peaks are produced at the same frequencies following the same pattern for low and high metal concentrations, demonstrating the high potential of the cost-effective and practical miniVNA tiny.

More research is required for evaluating, *in situ*, (1) the minimum concentration that can be detected, (2) at which concertation sensors start to deviate from linearity and (3) the effect of any possible interference in the freshwater, such as the presence of organic materials and any anion variations.

### 4.5. Results from the Continuous Measurement of a Tracer in Freshwater

Considering the infeasibility to measure, *in situ*, “unexpected” increases in trace metal contamination in water and the return to the baseline spectrum when the concentration decreases using microwave sensors, this was experimented using a slug salt injection.

After having evaluated the feasibility to detect changes in “salt”, such as NaCl and NaBr, using planar microwave IDE sensors in laboratory analysis with non-portable and portable VNAs [102], and sensors probing the water, a field trial was performed in the Nant Cwmnewyddion (Wemyss mine) for measuring the “unexpected” variation in a water parameter and the return to the baseline spectrum. 

**Figure 12:**
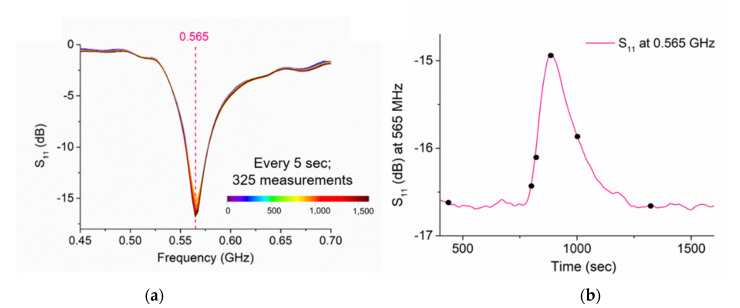
(**a**) Results from an experimental trial of a slug injection of NaCl using lacquered sensors probing the Nant Cwmnewyddion water measured every 5 s for 1625 s (27 min), demonstrating the feasibility of measuring, *in situ* and in real time, the variation in a water parameter (**a**); (**b**) the variation in the S_11_ response at 0.565 GHz by time and its return to the baseline once the concentration decreases.

For this experiment, performed in July 2018, 250 g of NaCl was dissolved in 10 L of river water (T = 20 °C). Its EC measured in the bucket was 36.6 mS/cm. This solution was “gulp” injected in the river and *in situ* measurements were performed ~100 m downstream (as illustrated in Figure 7 in Materials and Methods) using (i) a conductivity meter and (ii) a lacquered eight-pair IDE sensor connected to a mini VNA tiny, and a laptop as the output device. Measurements were performed every 5 s with both sensing devices for 27 min. The selected part of the spectral responses, between 0.45 and 0.70 GHz, is illustrated in Figure 12a, which comprises 325 S_11_ measurements (every 5 sec for 1650 sec); 0.565 GHz is the frequency that has been selected as being able to continuously monitor the variation in “salt in water”. The S_11_ response by time at 565 MHz is plotted in Figure 12b. The black dots in the graph are some selected instants where data are compared with EC and NaCl in Table 5. The NaCl concentration was measured successively using correspondence measurement between EC and g/L of salt in the lab with calibration curves developed on the same water samples. The variation in the salt concentration matches with both EC and S_11_ parameters, which is in line with the increase and decrease in the salt concentration at each instant. The S_11_ increases with the increase in EC and salt concentration.

This experiment has demonstrated the feasibility to measure the “unexpected” variation in a water parameter and its return to the baseline spectrum using microwave technology and planar sensors. The development of a sensing platform that is able to detect both NaCl and trace metal variations in complex catchments can provide strong support for researchers that work on fully characterising polluted areas and set priorities for remediation actions [29]. Using microwave measurement for real-time variations in both flow and metals could help to successfully manage polluted areas, in the UK and worldwide.

### 4.6. Future Strategies for Effective Applicability

This novel methodology offers the potential to reduce analytical costs by combining wireless sensing systems to provide continuous, *in situ* remote monitoring, thus facilitating the targeted treatment of mining-affected waters. The microwave sensors can be connected using a microcontroller-based system for measuring S_11_ and analysing the data. Then, results can be integrated with “risk ranking”, for ranking mining-impacted freshwater that threatens the environment [103]. 

The microwave sensors presented in this work can be the prototype for a smaller, portable online system capable of monitoring pollution in mining areas. This sensing technology could ensure continuous verification of water quality and safety and provide an immediate warning of contamination variations or water quality guideline threshold breaches. It could also offer the ability for *in situ*, real-time monitoring and reporting of water quality over large, remote geographical areas. 

Moreover, a dense wireless system of sensors can be deployed in mining-impacted catchments, offering the potential to reduce these costs considerably, as well as providing more useful, continuous monitoring capabilities by giving an accurate idea of the changes in water quality in real time. 

### 4.7. Summary of Contributions

In this work, progresses in microwave spectroscopy for liquid sensing are described using a novel approach based on submergible microwave sensors for monitoring trace metals. This is a fast-evolving field that is expected to grow exponentially due to constant advances in sensors and signal analysis [104]. As with microwave gas sensing [57], important features such as selectivity, sensitivity and long-term stability are not yet satisfactorily reported for water quality contaminants in water samples. Mostly, researchers worldwide have demonstrated the differentiation between laboratory-prepared liquid samples (mostly % mixtures water/alcohol) due to permittivity variations and consequent frequency shift, combining simulations and experiments at room temperature and obtaining selectivity in separated samples [105,106,107].

This research has contributed novel results for accomplishing innovative devices based on microwave spectroscopy, for *in situ* and real-time monitoring of water resources. Planar sensors can be used in the real environment and can offer a low-cost solution to monitor specific changes in water quality. The sensing platform can then be coupled with appropriate microcontrollers and wireless hardware, enabling remote operation in mining-affected rivers for true online monitoring [107,108].

## 5. Conclusions

This work describes a novel strategy that has been researched, developed and tested to meet the challenge of detecting real-time variations in toxic trace metal contamination in mining-impacted waters, at low cost and *in situ*. Currently, no method can guarantee continuous monitoring of water resources and evaluate real-time changes in water quality. Microwave spectroscopy is a low-cost sensing technology that allows a real-time and inexpensive response as soon as a material is in contact with the EM waves through a sensing structure. This work has demonstrated, for the first time, the practicability of characterising, *in situ*, different polluted waters, in the same or diverse mining areas, using submergible IDE sensors. The adapted eight-pair IDE sensor was able to quantify the contamination level at three resonant peaks, 0.05, 0.44 and 0.76 GHz. This work offers the basis for the further development of specific planar sensors which can classify and compare *in situ* and real-time polluted mining-impacted water. 

In conclusion, microwave spectroscopy and submergible planar sensors can help to monitor, identify, characterise and risk rank freshwater quality.

## Figures and Tables

**Table 1:** Zn, Cu and Pb average concentration ranges in some water impacted by non-coal mines in Europe.

Country	Mining District	Zn (mg/L)	Cu (mg/L)	Pb (mg/L)	Reference
SP	Rio Tinto	56–420	24–240	0.1–2.4	[24]
IT	Montevecchio	0.25–1200	0.21–3.4	0.56–3.60	[14]
FI	Luikonlahti	1.6–4.1	0.003–0.5	-	[20]
NO	Løkken	30–50	5–10	-	
NM	Zletovo	0.06–26.11	0.03–1.05	<0.03–0.08	[25]
GE	Kupferschiefer	0.41–1.05	0.080–0.360	0.06–0.08	[26]
UK	Force Crag Mine	0.21–2.95	-	0.005–0.097	[27]
UK	Parys Mountain Mine	1–10	0.01–3.0	-	[28]
UK	Cwm Rheidol Mine	13.5	-	0.75	[29]
UK	Afon Twymyn	0.01–1.7	<0.030	0.01–0.4	[30]
UK	Nant y Bai Lead Mine	0.5	-	0.28	[31]
UK	Parc Lead-Zinc Mine	0.27–0.34	-	0.38–2.60	[32]

(SP—Spain; IT—Italy; FI—Finland; NO—Norway; NM—North Macedonia; GE—Germany; UK—United Kingdom).

**Table 2:** Example of resonant cavities and their tested application for LUT measurements.

Sensing Structure	Specification	Tested LUT	References
Resonant cavity	Cylindrical	Water hardness (Ca^++^)	[69]
Resonant cavity	Cylindrical	Nitrates	[70]
Resonant cavity	Cylindrical	Silver material	[71]
Resonant cavity	Cylindrical	NaCl, KMnO_4_, methanol	[68]
Resonant cavity	Cylindrical	Gas–liquid two-phase flow regime	[72]
Resonant cavity	Rectangular	Drip loss	[60]
Resonant cavity	Rectangular	Nitrates and sulphites	[73]

**Table 3:** Examples of planar structures and their tested application for liquid analysis.

Sensing Structure	Specification	Tested LUT	References
Coplanar waveguide	With interdigital capacitor-loaded electric-LC resonators	Nitrate and phosphate	[78]
Planar multiband sensor	Split-ring resonators (SSRs)	Glyphosate (herbicide)	[79]
Planar sensor	Double-sided split-ring resonator (DSS-SRR)	Alcohols and water	[80]
Planar sensor	SSR	Glucose in water	[81]
Planar sensor	Complementary split-ring resonator (CSRR)	Water and ethanol	[82]
Planar sensor	E&C shape	Glycogen	[63]
Flexible planar sensor	IDE	NaCl, KCl, MnCl, CuCl	[83]
Planar sensor	Microstrip line and an SRR	NaCl, KCl, CaCl_2_, MgCl_2_ and Na_2_CO_3_ in water	[84]
Planar sensor	IDE	Tetraselmis suecica	[85]
Planar sensor	IDE	Lincomycin and tylosin antibiotics	[86]
Planar sensor	Double quadratic shape	Ag nanoparticles in DW	[87]
Planar resonator	with 3D printed channel	Ethanol and DW	[88]
Planar sensor	IDE + microfluidic	DW and alcohol; DW and NaCl	[89]

**Table 4:** Statistical features, mean, SD and CV for a collected water sample (NC) spiked with Cu standard addition (+1.25 mg/L).

	S_11_ (dB)	S_11_ (dB)	S_11_ (dB)	SD	SD	SD	CV	CV	CV
Cu mg/L	0.05 GHz	0.44 GHz	0.76 GHz	0.05 GHz	0.44 GHz	0.76 GHz	0.05 GHz	0.44 GHz	0.76 GHz
0	−0.73203	−3.53312	−13.8452	0.07184	0.00707	0.02048	9.813805	0.200106	0.147922
1.25	−0.80696	−3.58873	−14.3714	0.21113	0.01857	0.01867	26.16363	0.517453	0.129911
2.50	−1.14271	−4.04695	−11.4981	0.21113	0.01004	0.23926	18.47625	0.248088	2.080858
3.75	−1.65849	−4.55473	−11.4853	0.21113	0.01655	0.01482	12.73025	0.363359	0.129035
5.00	−2.21526	−5.25246	−10.0652	0.21113	0.01469	0.00957	9.53071	0.279678	0.095081
6.25	−2.68925	−5.82367	−9.5899	0.21113	0.01927	0.01965	7.850888	0.330891	0.204903

**Table 5:** Variation in EC and S_11_ signal by time due to change in salt *in situ* and in real time and the return to the baseline spectrum. NaCl was successively measured by applying conversion equations.

Time (sec)		EC (µS/cm)	S_11_ at 565 MHz (dB)	NaCl (mg/L)
0	baseline	113.6	−16.60	0
780	salt detected	124.2	−16.43	0.004
820	salt increasing	235	−16.08	0.005
880	peak of salt	379	−14.93	0.131
995	decrease of salt	226	−15.86	0.056
1320	return to the baseline	110.0	−16.65	0

## Appendix A

**Table A1:** Illustrates physicochemical parameters (EC, pH, T) for mining-impacted water samples measured in both field and lab, when were performed microwave measurements. Trace metals concentrations analysed using ICP-MS and/or ICP-OES for the collected mining-impacted water samples are also described.

Sample	Mining Area	*In Situ*			In Lab			Trace metals					
		EC	pH	T	EC	pH	T	Zn	Cu	Pb	Cd	Fe	Mn
		µS/cm		°C	µS/cm		°C	µg/L	µg/L	µg/L	µg/L	µg/L	µg/L
NC	Wemyss mine	115	6.26	19.6	118.0	6.33	19.0	2935.171	<0.0001	48.01	<0.0001	5.61	<0.0001
PM (A)	Parys Mountain	3200 *	2.44 *	13.4	3300 *	2.45 *	19.1	10,640.05 *	9657.249 *	20.897	116.021	44,115.08	10,153.33
PM-1	Parys Mountain	1634 *	2.78 *	10.2	1715 *	2.46 *	19.1	12,453 *	7029.993 *	888.582	69.632	86,232.68	52.048
PM-2	Parys Mountain	7.570 *	1.80 *	10.8	6030 *	1.94 *	19.0	13,542.03 *	25,008 *	210.852	48.668	511,223.3	2594.671
PM-W	Parys Mountain	249	5.9	18.9	280	6.2	19.0	1231.423	1131.653	12.342	20.993	3210.617	300.582
NYB-1	Nant y Mwyn	87.9	6.62	18.7	87.5	7.12	19.2	951.624	8.116	417.507	5.225	46.708	3.752
NYB-2	Nant y Mwyn	76.1	6.67	14.7	77.0	6.84	19.0	754.438	7.856	331.159	3.915	70.577	8.076
NYB-R	Nant y Mwyn	109.3	6.60	14.8	110.2	6.18	18.9	1729.217	68.981	1970.48	10.685	148.068	8.753
WW-1	Leadhills mine	132.0	7.46	10.8	157.7	7.22	19.0	273.813	3.375	61.949	2.88	54.031	17.00

*: values which should indicate extremely polluted water. All values measured using a ICP-MS were accurate (DL < 0.0001 µg/L for all measurements). All values measured using a ICP-MS were accurate precise (CV < 0.5%).
